# Effect of Acupressure on Fatigue in Women With Multiple Sclerosis

**DOI:** 10.5539/gjhs.v7n4p375

**Published:** 2014-01-25

**Authors:** Farideh Bastani, Marzieh Sobhani, Hormat Sadat Emamzadeh Ghasemi

**Affiliations:** 1 School of Nursing and Midwifery, Department of Geriatrics Nursing, Iran University of Medical Sciences, Tehran, Iran; 2 School of Nursing & Midwifery, Iran University of Medical Sciences, Tehran, Iran; 3 School of Nursing and Midwifery, Tehran University of Medical Sciences, Tehran. Iran

**Keywords:** multiple sclerosis, fatigue, acupressure, women health, symptom

## Abstract

**Introduction:**

Multiple sclerosis (MS) is the most common cause of progressive neurological disability. The prevalence of MS is much more common in women than men. The women are exposed to a variety of symptoms including fatigue. Acupressure is a noninvasive procedure that can be used to control symptoms including fatigue. The aim of the study was to evaluate the effect of acupressure on fatigue in women with multiple sclerosis.

**Methods:**

A randomized clinical trial was conducted on 100 women with MS at Tehran MS Association. The subjects were equally allocated to experimental group and a placebo group (50 women per group) by blocking randomization method. The experimental group were received acupressure, at the true points (ST36, SP6, LI4) and the placebo group, were received touching at the same points. Fatigue was measured by a Fatigue Severity Scale (FSS) in the groups at immediately prior to, two and four weeks after the beginning of the intervention. The data was analyzed using descriptive and inferential statistics by SPSS version 17.

**Results:**

The findings indicated no differences in demographic characteristics and the severity of fatigue at the baseline in two groups (p=0.54). But there were significant reductions of the mean score of fatigue in the experimental group compared to the placebo group immediately, two and four weeks after the intervention respectively (p=0.03, p≤0/001, p=0.04).

**Conclusion:**

According to the findings, the study provided an alternative method for health care providers including nurses to train acupressure to the clients with MS to managing their fatigue.

## 1. Introduction

Multiple Sclerosis (MS) is the most prevalent neurological disease (Mao & Reddy, 2010). It is characterized by lesions and scarring of the protective myelin sheath of the central nervous system (CNS), leading to neuronal damage and axonal loss (Burgess, 2002; Calasanti & King, 2007). It is more common in women than in men (Schwendimann & Alekseeva, 2007). According to a study in Tehran, the prevalence of MS in Tehran has been estimated to be 51.9 per 100,000 people in 2010 (Sahraian et al., 2010). Multiple Sclerosis is unpredictable and is one of the major diseases affecting patients’ quality of life (Janardhan & Bakshi, 2002). The course of the disease is uncertain and the clients with MS may face physical problems including muscle weakness, bladder and bowel dysfunction, problems with speech, and vision (Schapiro, 2007) and also hidden difficulties such as fatigue (Burgess, 2002; Calasanti & King, 2007). The patient’s fatigue might be extremely debilitating and often causes one to have a sedentary lifestyle i.e. sitting, lying down or sleeping. Moreover, it has a great influence on all aspects of life, particularly on occupation, daily life activities, social relationships and self-care activities (Samuels & Ropper, 2010).

Currently, many pharmacologic and non-pharmacologic methods are used to relieve fatigue. However, pharmacologic approaches such as Amantadine to control fatigue in women with MS may have side-effects including insomnia, increased constipation and increased urinary retention for them (Induruwa, Constantinescu, & Gran, 2012). Therefore, it seems that using non-pharmacological methods is necessary in order to reduce the side effects. During recent years, the attention of many patients including MS patients has been drawn to non-pharmacological methods which are known by the name of complementary alternative therapies (CAT) (Kochs, Wegener, Sühnel, Voigt, & Zettl, 2014). Acupressure as a CAT has been reported to be useful in symptom management in a variety of patient populations (Chen & Chen, 2004). It is a non-invasive, cost-effective technique, with no side-effects (Cho & Hwang, 2010). The advantage of this method is that it is easy to use and learn, and therefore it can be applied by the patients, themselves (Esmonde & Long, 2008). It is noteworthy that there have been no cases of incompatibility between acupressure and other therapeutic methods; therefore, acupressure can be used simultaneously with other methods. The principles of acupressure are similar to acupuncture except that no needle is used, and the acu-points are stimulated using fingers or special tools (Polit & Beck, 2003). In the light of the limited research on fatigue reduction interventions in women with MS the present study was undertaken to investigate the effect of nurse-provided acupressure on fatigue in women with MS.

## 2. Method

**Figure 1:**
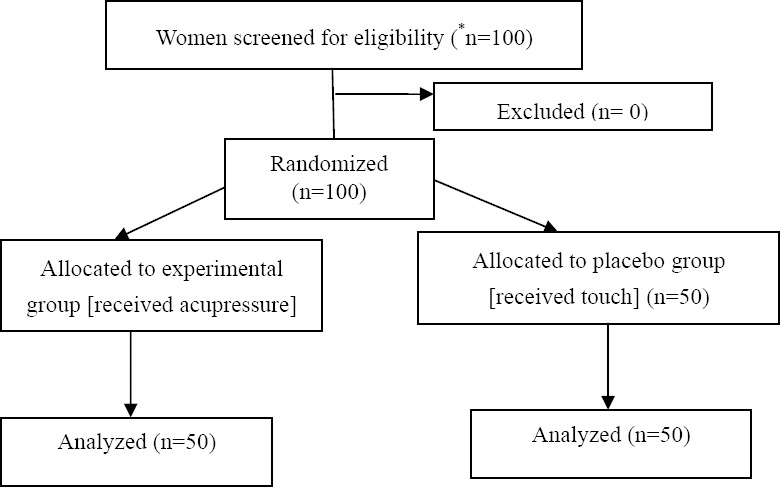
Consort flow diagram indicating patient screening, intervention allocation, and analysis *n= number of patients

This study was a randomized clinical trial (RCT) with a prospective pre-post experimental design. This RCT was conducted on 100 women with MS at “Tehran Multiple Sclerosis (MS) Association”. The subjects as the member of the MS Association were selected with continuous sampling method. The subjects were equally allocated to the experimental group and the placebo group (50 women per group) by blocking randomization method. The sample size was estimated according to the power analysis and considering a normal distribution for the main primary outcome measures. In the study, the power, effect size, and alpha were set as 0.80, 0.20, and 0.05, respectively (Yeung et al., 2012), based on the results of a previous study (Sobhani, 2012). The flow of patients through the trial is presented in Figure 1.

Approval for the study was given by the Ethics Committee of Tehran University of Medical Sciences in accordance with the ethical standards laid down in the 1995 Declaration of Helsinki. Written informed consent was obtained from the participants prior to their inclusion in the study. The participating women were also given verbal information before taking their informed consent. The inclusion criteria were: (a) age at least 18 years, (b) stable vital signs, (c) no scar, lesion, scratch or deformities on the skin of selected areas (d) being literate, (e) complaining of fatigue (assessed by the Fatigue Severity Scale [FSS] with the score of 5 and over, (f) no history of smoking, substance or sedatives use and (g) not pregnant. The exclusion criteria were lack of the subjects’ willingness to continue participation in the trial for any reason, such as complications, or known serious physical or mental diseases during the trial. Also, the women who had not feeling of warmth, heaviness, or numbness during applying acupressure on the points LI4, ST36, and SP6 for any reason were excluded from the study. The primary outcome as the fatigue severity was measured immediately prior the intervention and immediately, two and four weeks after the intervention, to evaluate the effect of nurse-provided acupressure on fatigue in women with MS between August 2011 and October 2011.

The experimental group were received acupressure, at the acupoints (ST36, SP6, LI4) and the placebo group, were received touching at the same points in the first session. The duration of each session of the intervention was 3 minutes bilaterally, for each group. In other words, the acupressure intervention, i.e. pressure on the acupoints, was conducted for three minutes (several cycles including 10 seconds consecutive pressure and 2 seconds rest) on each of the mentioned points, and then this was repeated for the opposite side of the body. This procedure took 18 minutes for each intervention per day. During training session the researcher demonstrated the procedure in one part of the patient’s body, and asked her to do the same herself on the other side of the body. The training was over when the correct practice by the patients was ensured. It was explained to the patients that the accuracy of the points or channels are confirmed by the client feeling warmth, heaviness, or numbness in that special areas. These procedures were also performed in the placebo group but by touching rather than pressing the required three points that were similar to the experimental group. Also the placebo group was not given the pamphlet.

After the completion of the intervention on both sides of the body at the first session, the women completed FSS again, and it was considered as the immediate post- test intervention. Then the groups were trained and taught to perform the same procedure for themselves (as Self-administered acupressure) at the same points twice daily for two weeks, at home. The researcher who had a certificate of applying acupressure and she was taught under the supervision of an acupuncturist and acupressure specialist, was responsible for the education. The first individual training session was implemented in a conference room at the MS Association for each subject by the researcher for 35 minutes with a face-to-face education.

**Figure 2:**
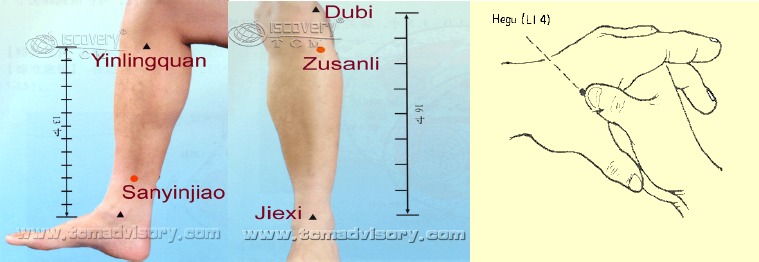
The locations of the acupoints LI4, ST36 and SP6

The oral educational session was supplemented with pamphlets, demonstrating the technique of pressing the true acu-points with accurate duration, showing posters and slides by PowerPoint Software for the experimental group after providing full explanation about the research objective. The same researcher educated the placebo group about the procedure of touching at the same acu-points (except providing pamphlet). One of the required acupoints was L.I.4 or Hegu channel which is the major painless relaxant point. It is located on the dorsum of the hand, between the 1st and 2nd metacarpal bones, in the middle of the 2nd metacarpal bone on the radial side, when the thumb is fully extended, i.e. the bones of the thumb and the first finger. The other is the ST36 point or Zusanli which is to the lateral side of the middle toe, 3 individual cun below the genu, i.e. on the anterior lateral side of the leg, one finger breadth (middle finger) from the anterior crest of the tibia, which is one of the most effective locations for reduction of fatigue and a general balance point. The last point is SP6 or Sanyinjiao, which is on the medial side of the leg, 3 cun above the tip of the medial malleolus, posterior to the medial border of the tibia, is one of the major and most commonly used channels (Molassiotis, Sylt, & Diggins, 2007) which is shown in Figure 2.

Fatigue as a primary outcome, was measured by the Fatigue Severity Scale (FSS) in the groups at immediately prior to, and immediately two and four weeks after the beginning of the intervention. The fatigue severity scale (FSS) measures the patient’s ability to function with nine statements each of which are scored from 1-7 in Likert scale, by classifying them as 1 (completely disagree) to 7 (completely agree). The final score is calculated by averaging the sum of responses divided by nine. Therefore, the mean score was used to compare the severity of fatigue in the two groups. The scale is one of the highly reliable tools to investigate fatigue, and to only evaluate the concept of fatigue. This tool evaluates fatigue in MS patients more quickly compared with other tools; that is to say that the obtained score is fully compatible with the rate and severity of fatigue. The reliability of FSS has been measured through Cronbach’s alpha with coefficient 0.77 for patients with MS (Learmonth et al., 2013).

In order the patients do not forget applying the intervention twice daily for two weeks; they were given a reminder timetable to mark the schedule each time after implementing the intervention (in the morning and afternoon). Thereafter, during the two weeks intervention, the researcher followed-up their procedures through phone calls every day. In addition, the patients were given two copies of the questionnaire of FSS to complete them; one after the end of the intervention at the end of the second week (14th day), and the other, at the end of the forth week (28th day). Totally, FSS was completed four times including pre-first intervention and immediately after the first intervention (with the presence of the researcher) and at the end of day 14 and day 28. Besides, in order to avoid any possible and potential bias, implementation of the intervention was done only by one researcher. Finally, at the end of the trial and the post- phases, the data were analyzed through SPSS for Windows version 17.0 (SPSS, Inc., Chicago, IL, USA) and applying chi square, Fisher’s exact-test, independent t-test and repeated measure ANOVA; hence, the two groups were compared to each other.

## 3. Results

The study results showed that there were no significant differences between the two groups in terms of age, duration and recurrence of the disease (Table 1).

**Table 1:** Women’s Demographic and Clinical Characteristics by Group

Variables	P-value	M(SD)	

		Acupressure (n=50)	Placebo (n=50)
Age (year)	(P=0.98)	31.88(6.21)	31.90(6.33)
Duration of MS (year)	(P=0.22)	2.86(1.27)	3.16(1.18)
Frequency of MS relapse (year)	(P=0.90)	1.58(0.75)	1.60(0.90)

The groups were similar in terms of other demographic variables including education, marital status, occupation and medication (amantadine) with regard to fatigue. Statistically, there was no significant difference between fatigue severity in the experimental and placebo groups before the intervention. But, based on the independent t-test, there was a significant difference between the two groups in terms of fatigue severity immediately, two, and four weeks after the intervention (Table 2).

**Table 2:** Comparison of the Mean Fatigue Scores in the Groups in Different Phases

Groups	Phases			

	Pre- intervention	immediately after intervention	2-week after intervention	4-week after intervention
Experimental	88.5(55.0)	44.5(90.0)	44.4(84.0)	65.5(83.0)
Placebo	82.5(54.0)	79.5(56.0)	89.5(58.0)	95.5(59.0)
P. value	0.60	0.02	0.001	0.004

Fatigue severity was lower in the experimental group compared to the placebo group. In addition, repeated measures ANOVA (with the Bonferroni method for pairwise comparison) were used to compare the mean scores of fatigue severity during the four phases, i.e. before, immediately, two weeks and four weeks after the intervention which the differences were statistically significant for the three phases at post-test compared to the pre-intervention phase (P ≤ 0.001). However, such a trend was not evident in the placebo group in the study. Also, no complication occurred during the acupressure.

## 4. Discussion

The present study generally aimed to evaluate the impact of acupressure on the severity of fatigue in women with MS. In this study, there were no significant differences regarding demographic variables and also severity of fatigue in two groups at pre-intervention (p≤0.05). But at post- test, the results showed significant improvement of the mean scores of fatigue in the experimental group compared to the placebo group at immediately, two, and four weeks after the intervention. The result was consistent with the finding of a systematic review study, indicated that use of acupressure significantly effect on improving symptoms including fatigue in the acupressure group (Lee & Frazier, 2011). This study focused on fatigue which is mostly underdiagnosed and poor management that contributes to increased symptom burden and poorer quality of life in patients (McKeown, Porter-Armstrong, & Baxter, 2003) As there are scares studies concerning the impact of acupuncture or acupressure on fatigue of patients, it should be emphasized that the symptom of fatigue has also an adverse effect on quality of life and life satisfaction of the patients with MS (Y. H. Kim, H. J. Kim, Ahn, Seo, & S. H. Kim, 2013) therefore; non-pharmacological methods to improve patients’ fatigue could be an important approach regarding holistic nursing care. Zick et al. believe that acupressure as a branch of acupuncture could be a useful therapy for improving fatigue as well as sleep quality and quantity in patients with chronic diseases (Zick et al., 2010). Since acupressure is a method of complementary therapy, the results are congruent with the result of a systematic review study that revealed that exercise training as a complementary therapy is effective for improving fatigue, and health-related quality of life in patients with chronic diseases (Latimer-Cheung et al., 2013).

Though differences in intervention type and in acupressure treatments, study population, length of study and duration/frequency of acupressure intervention, acupoint locations, fatigue scales, and use of acupuncture rather than acupressure should all be considered in comparisons of the findings. Moreover, no participant stopped their acupressure or withdrew from the study as a result of these effects. Because the relative homogeneity of our study sample of women predominantly literate and in their reproductive age, may be a main reason that our participants responded more favorably to acupressure training than observed in other studies. This study is unique as the findings revealed that acupressure at the point (ST36, SP6, and LI4) can reduce the fatigue of the women with MS, in the acupressure group compared to the placebo. Acupressure techniques can be easily taught to patients so that they can manage relaxation themselves (Guerreiro da Silva, Nakamura, Cordeiro, & Kulay, 2005). In an attempt to offer comfort and improvement of fatigue to patients, nursing is becoming increasingly involved in offering complementary-alternative therapies as part of caring-healing process in comprehensive patient care (Maa et al., 2007). Acupressure is one such modality that is being increasingly used by both medical and nursing professionals.

There are some limitations that should also be noted. This study focused on a self-report questionnaire for assessing subjective aspect of fatigue and also not investigating the effect of acupressure for a longer period more than four weeks. Therefore, it may be possible that evaluation of fatigue with more prolonged period acupressure might lead in more conclusive results. Further research is also necessary to test the benefits of acupressure with different duration and gender on symptom management including fatigue with a larger sample. Therefore, the results, may have limited generalizability to other population such as male patients with MS or even other general population. On the other hand, we should also mention that consecutive follow-ups after the first session of the acupressure training and phone call follow-ups, as a reminder timetable to the groups, could be the strengths of this study in terms of confirming the regular performance of self-administered acupressure in the participants and not losing the subjects. Therefore, further research to assess the utilization of acupressure for reducing fatigue and promote the comfort of the patients (males and females) with MS is strongly recommended.

In conclusion, follow-up tests indicated there were significant differences of the mean scores of fatigue between the acupressure group and the placebo group (p<0.001). Acupressure is a non-pharmacological, cost-effective and simple technique, which is applicable anywhere and anytime. Therefore, the study provided an alternative method for health care providers including nurses to train acupressure to the clients with MS to managing their fatigue.
